# Cardiac Surgery During and After the Pandemic: A Retrospective Analysis of UK Trends and Outcomes

**DOI:** 10.1093/ejcts/ezaf246

**Published:** 2025-07-28

**Authors:** Tim Dong, Norman Briffa, Pradeep Narayan, Jeremy Chan, Gianni D Angelini

**Affiliations:** Bristol Heart Institute, Bristol University, Bristol, BS2 8HW, United Kingdom; Cardiothoracic Surgery Department, Sheffield Teaching Hospitals, Sheffield, S5 7AU, United Kingdom; Rabindranath Tagore International Institute of Cardiac Sciences, Narayana Health, Bangalore, 700099, India; Bristol Heart Institute, Bristol University, Bristol, BS2 8HW, United Kingdom; Bristol Heart Institute, Bristol University, Bristol, BS2 8HW, United Kingdom

**Keywords:** heterogeneity, multi-modality, statistical trends, risk analysis, cardiovascular care, surgical quality

## Abstract

**Objectives:**

The full extent of the COVID-19 pandemic’s impact during different phases of the pandemic and the recovery of cardiac surgical services in the United Kingdom have not been comprehensively assessed. This study aims to evaluate these disruptions’ impact and immediate recovery on delivering adult cardiac surgical care in the United Kingdom.

**Methods:**

The periods investigated were divided into pre-lockdown, first lockdown, first relaxation, second lockdown, second relaxation, third lockdown, and post-lockdown (recovery). Changes in surgical practice, early and mid-term clinical outcomes, and hospital readmission were analysed using various metrics across different time periods.

**Results:**

Coronary artery bypass grafts were the most performed procedure across all time periods, with the average number of urgent and emergency increasing compared to the pre-pandemic period. Aortic valve replacement was the next most frequent, followed by combined aortic valve and coronary artery bypass surgery. However, those procedures remained predominantly elective across all periods. There was a significant change in 30-day mortality rates across the pandemic phases (*P* < .001), with higher mortality observed post-pandemic. There was a gradual increase in the waiting times for elective and urgent surgeries from January 2018 to March 2022. Patients who had surgery before March 2020 had a significantly lower hazard of mid-term mortality than those who were operated on after this period (HR, 0.638; 95% CI, 0.5875-0.6921). All procedures showed gradual recovery across the pandemic periods following an initial decline at the beginning of the pandemic.

**Conclusions:**

COVID-19 had a significant negative impact on adult cardiac surgical case mix and volume and has not recovered to the pre-pandemic levels. This work, we believe, is important for policymakers, healthcare providers, and patients, as it offers insights into the challenges faced by a critical healthcare sector during a global crisis and highlights potential avenues for improvement.

## Graphical abstract

**Figure:**
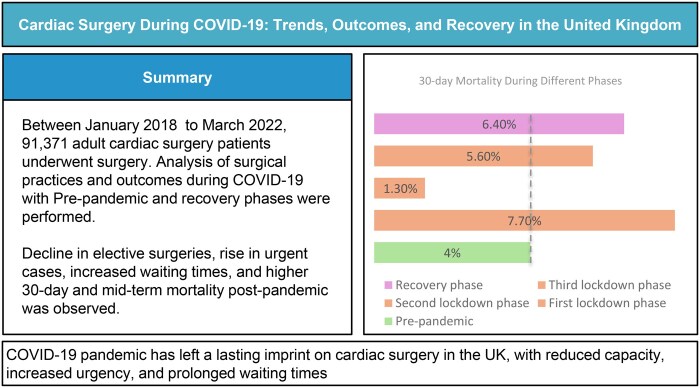

## INTRODUCTION

The COVID-19 pandemic caused unprecedented upheaval in global healthcare systems, with cardiac surgery experiencing significant disruptions due to its resource-intensive nature and high-risk patient population. In the United Kingdom, initial responses involved postponing elective procedures and reallocating resources, leading to immediate reductions in surgical activity.^1^^,^^2^ Early studies revealed concerning trends, including increased mortality risk for patients with COVID-19 undergoing cardiac surgery and the potential for nosocomial transmission within cardiac units.^3^^,^^4^

Further research during the start of the pandemic in 2020 highlighted its multifaceted impact on cardiac surgical services, including significant reductions in surgical volume across various procedures, namely valve surgery and coronary artery bypass grafting (CABG).^5^^,^^6^ These studies highlighted the need to better understand post-operative outcomes in cardiac surgery cases and monitor future trajectories. The pandemic also exacerbated existing workforce challenges, with staff shortages and redeployment contributing to delays in care and potential burnout among healthcare professionals.^7^^,^^8^

While these studies shed light on the acute effects of COVID-19, very little has been published on the effect of the pandemic on trend as well as early and mid-term outcomes after cardiac surgical interventions. This study aimed to evaluate the trends during the pandemic of COVID-19 lockdowns and relaxations, as well as the subsequent recovery, on surgical practice and outcomes in adults undergoing cardiac surgery, by comparing key metrics across different pandemic phases.

Data from multiple national registries were used.

## METHODS

### Study design and setting

This was a retrospective cohort study to analyse the impact of COVID-19 on the variability in presentations, practices and early (within hospital) to mid-term (following discharge but ≤5 years post-operation) outcomes of patients undergoing adult cardiac surgery at all NHS hospitals in the United Kingdom. The COVID-19 induced lockdown phases between January 1, 2018 to March 31, 2022 were divided into 5 distinct time periods as follows:

1. January 1, 2018 to January 1, 2020 (pre-lockdown period)
2. March 23, 2020 to November 4, 2020 (from first lockdown to its relaxation)
3. November 5, 2020 to January 5, 2021 (from second lockdown to its relaxation)
4. January 6, 2021 to June 21, 2021 (from third lockdown to its relaxation)
5. June 22, 2021 to March 31, 2022 (post-lockdown or recovery phase)

The periods between January and March 2020 were excluded to provide an approximately balanced time frame of 2 years for the pre-pandemic period and approximately 2 years for the post-pandemic period.

### Population and data source

The study population included all first-time adult cardiac surgeries with a UK National Adult Cardiac Surgery (NACSA) database record during the study period. Records were excluded if they failed the following data quality checks: absence of a valid patient ID for data linkage, missing operation date, Lower Super Output Area (LSOA) outside England, age at time of operation younger than 17 or older than 90 years, or if the patient was not alive on the date of operation. Additional quality checks were applied to remove any remaining implausible records, including missing or invalid date of death in Civil Registration of Deaths, date of death occurring before the date of birth, missing or invalid sex, date of birth or person identifier, or males with pregnancy codes.

The data sources used for this study include linked national electronic health records for England, accessed through NHS England’s Secure Data Environment service for England, via the BHF Data Science Centre’s CVD-COVID-UK/COVID-IMPACT consortium. The specific data sources include National Adult Cardiac Surgery Audit (NACSA), Primary care data from the General Practice Extraction Service (GPES), Data for Pandemic Planning and Research (GDPPR), Secondary care data from Hospital Episode Statistics: Admitted Patient Care (HES APC), HES-AE (accident and emergency), Outpatients (HES OP), and Critical Care (HES CC), COVID-19 testing data from the Second Generation Surveillance System (SGSS), including Pillar 2 testing, Secondary Uses Service (SUS), COVID-19 SARI-Watch dataset (formerly CHESS), the Office for National Statistics (ONS), as well as Civil Registration of Deaths dataset. A detailed description of other covariates as well as treatment of missing values are provided in the Supplementary Materials, Covariates and Dealing with Missingness section.

### Outcomes of interest

The primary end-point was defined as the difference in mortality risk between the pre-lockdown period and other pandemic periods. The following were defined as outcome measures of interest: trends in type, volumes and urgency of operative procedures; trend of acute episodes of cardiac condition requiring urgent admission in the 3-6 months prior to admission for surgery used as a surrogate for disease progression; early outcomes after cardiac surgery including mortality, stroke, need for prolonged cardiovascular or respiratory support, and total length of hospital stay. The waiting times for elective and urgent cardiac surgery were measured by the time (number of days per month) between the last cardiac catheterization and the surgery date (first procedure across spells). Mid-term outcomes in terms of survival (up to the end of the study, March 31, 2022) and hospital readmissions within the first 6 months (as defined by main cardiovascular reasons for readmission: ICD-10 diagnostic codes: [I10-15, I20-28, I30-52, I60-69] were also recorded; details are provided in **Table S1**). Non-cardiovascular reasons for readmission were excluded.

### Statistical analysis

Chi-squared test and Kruskal-Wallis test were conducted for the categorical and continuous variables, respectively. Due to the sample size in groups exceeding 5000, the Shapiro-Wilk test could not be used, and Quantile-Quantile (QQ) plots were used to assess against normality assumptions. Wilcoxon rank-sum analysis of waiting times for elective, urgent, emergency and salvage groups were conducted with the pre-lockdown values compared across other lockdown periods in each group. Since there is no referral variable available in the dataset, Z-test have been used to estimate the difference in mean percentage of the number of referrals (using surrogate date of last cardiac catheterization) for elective, urgent, emergency, and salvage groups with difference calculated using pre-lockdown values compared against other lockdown and relaxation period values in each group.^9^ Since the level of urgency, operative mortality risk and patients positive for COVID-19 were factors affecting indication bias for surgery during the pandemic, the following additional statistical analyses were conducted. The age (quartiles) and covid diagnosis adjusted mixed effects logistic regression (with hospital as the random effect) was used to estimate the ORs for (i) elective vs urgent, emergency, and salvage groups and (ii) post-procedure mortality any time within specified pandemic periods (yes vs no), comparing each pandemic period with pre-lockdown period. For the mortality analysis, we additionally adjusted for case mix using logistic EuroSCORE risk factors.

Perioperative mortality risk scores were obtained from the logistic EuroSCORE, from 2018 to 2022.^10^ Min-max normalization of each constituent variable of the model to improve comparability by scaling values to between 0 and 1.^11^ The mean of each normalized variable values was calculated for each year and plotted using cubic spline smoothing and compared to the spline for logistic EuroSCORE and their 95% confidence intervals. The cubic splines were analysed using the geom_smooth function of the ggplot2 package in R. For the change in number of acute episodes of cardiac condition requiring urgent admission in the 3-6 months prior to admission for surgery, the number of cardiac admissions for urgent cases were summed across the 3-6 months prior to surgery and fitted using a spline. The change in mid-term outcomes in terms of survival was visualized using the Kaplan-Meier curve with the log rank test for significance and mortality date from ONS. Cox proportional hazard regression models with and without confounders adjustments was fitted as a sensitivity test to compare the Hazard Ratios (HR) in periods before and after March 23, 2020, ie, before and after pandemic. The adjusted HR for subjects with covariate values $X_{i}$ compared to subjects with the reference covariate values $x_{i,\mathrm{ref}}$ is given by^12^^,^^13^:


(1) $${HR}_{i}(X_{i},x_{i,\mathrm{ref}})=\text{exp}(\beta_{i}X_{i}-\beta_{i}x_{i,\mathrm{ref}})$$

This analysis was performed according to a pre-specified analysis plan published on GitHub, along with the phenotyping and analysis code (https://github.com/BHFDSC/CCU007_05).

## RESULTS

**Figure 1.**
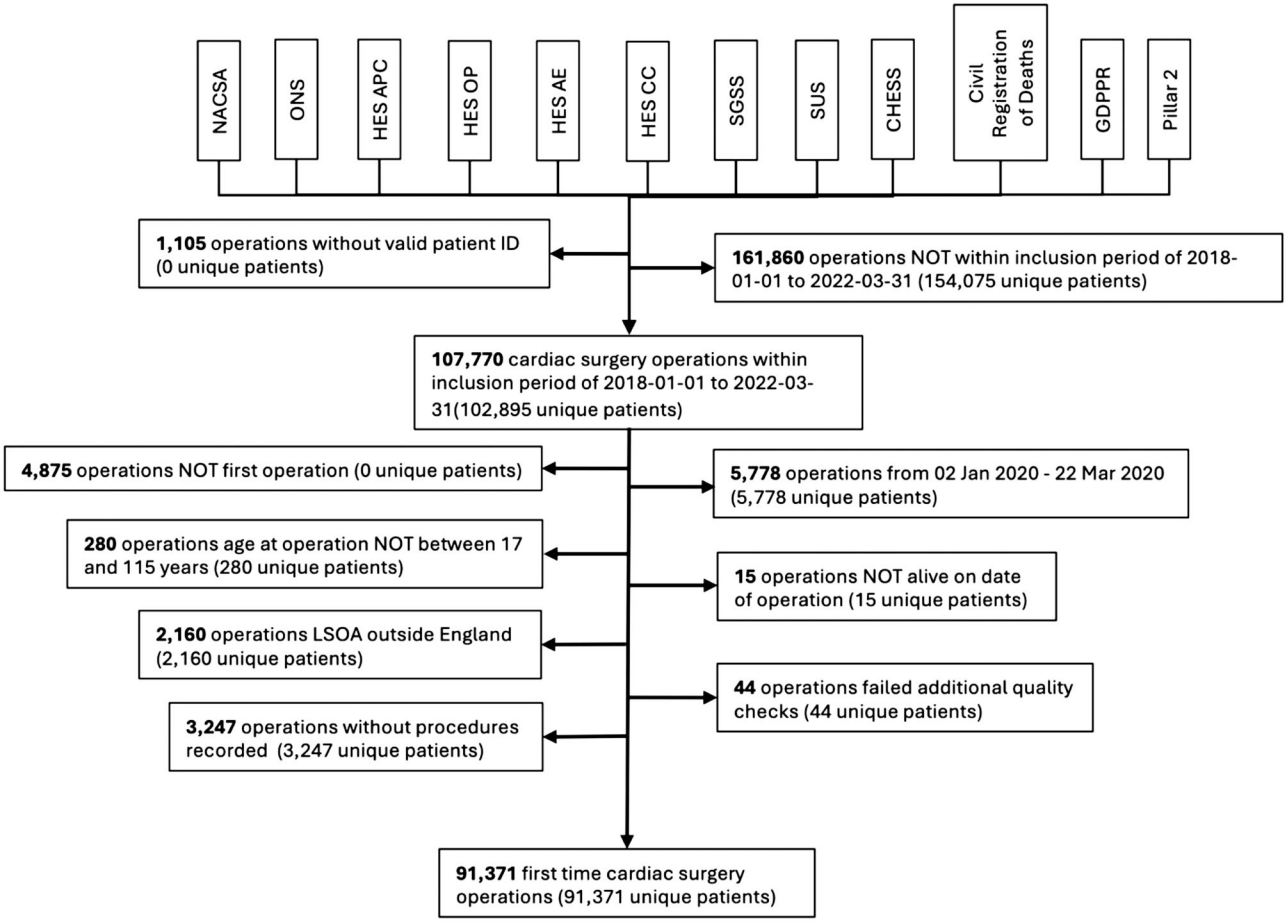
Flow Chart Adult Cardiac Surgery Operations from the Linked Datasets for Inclusion in Analysis

From January 1, 2018, to March 31, 2022, 107 770 patients underwent surgery. Following exclusion criteria, a total of 91 371 eligible patients were included in the analysis (**Figure 1**). There were 4.41% (4030/91 371) patients positive for COVID in the study.

### Trends in type, volumes, waiting times, and urgency of operative procedures

**Figure 2.**
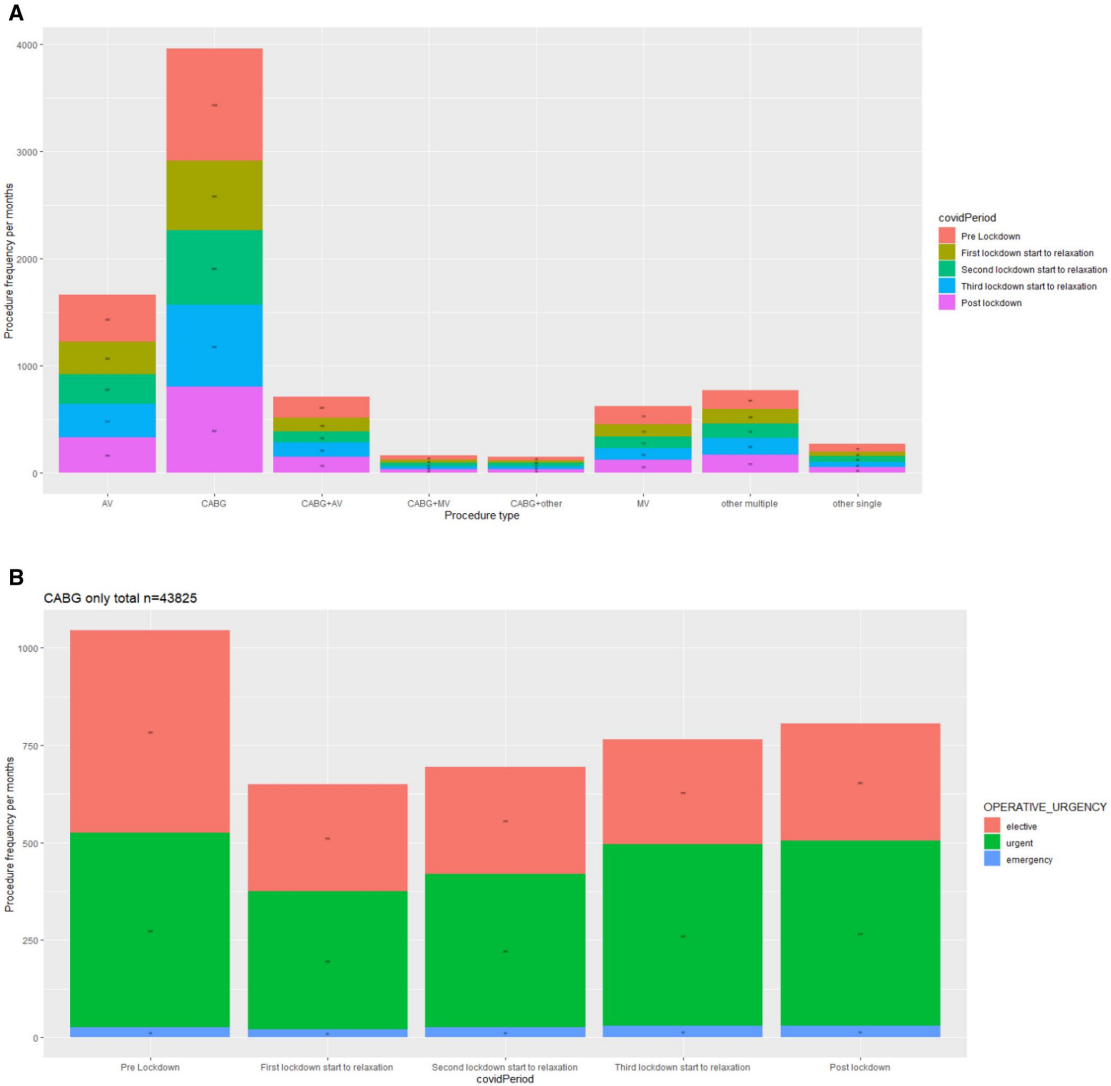
(A) Change in the types of procedure performed in each of the 5 periods. (B) CABG procedures subdivided by elective, urgent, emergency categories. Y-axis represents the average number of procedures per months

CABG was the most performed procedure across all time periods, with the highest average procedural frequency per month recorded in the pre-lockdown phase (**Figure 2A**). Aortic valve (AVR) procedure was the next most frequent procedure across all time periods, followed by CABG + AVR and mitral valve (MVR) procedures. There were relatively low frequencies of CABG+MVR and CABG+other procedures across all time periods. All procedures showed gradual recovery across pandemic periods following an initial decline after the beginning of the pandemic. Following an initial decrease from pre-lockdown numbers, there was an increase in the average number of urgent and emergency CABG and non-CABG procedures across the subsequent lockdown periods (**Figure 2B** and **Figure S1**). However, non-CABG procedures remained predominantly elective in nature across all time periods.

A gradual increase was observed in the waiting times for elective and urgent surgeries from January 2018 to March 2022 (**Figure S2**). In the pre-lockdown phase in March 2019, the peak waiting time was 193 days, which increased to 199 during the first lockdown period. There was a slight decrease in the waiting time during the second lockdown period, with a peak of 173 days, followed by a significantly increased waiting period of 216 days from June 2021 towards the end of the third lockdown. In the post-lockdown phase, the upward trend in the waiting time continued, with a peak of 202 days in February 2022.

A similar upward trend was noted for the urgent cases. In most months in the pre-lockdown phase, the waiting time for urgent cases remained below 40 days. However, in most months, the first lockdown period was above 40 days. In the third lockdown phase, the waiting time for urgent cases rose further above 50 days in most months. During the post-lockdown phase, the upward trend continued, with most months witnessing a waiting time greater than 40 days.

For the urgent group, although there were no changes in waiting time detected in the first and second lockdown periods, the median waiting time increased significantly by 2 days per month in the third and post-lockdown periods compared to pre-lockdown (*P* < .05; **Figure S3**). The waiting times were also increased significantly in the elective group in all pandemic periods except the second lockdown. Overall, there were no significant changes in waiting time for the emergency and salvage groups, other than some signs of an increase in the emergency group post-lockdown.

The number of cardiac catheterizations (as a surrogate of referrals) increased substantially during the pandemic period compared to the pre-lockdown period for elective and urgent procedure groups (*P* < .05; **Figure S4**). There was a clear trend towards increasingly greater mean number of urgent referrals in each subsequent pandemic period from the second lockdown onwards (**Figure S4**, urgent group).

### Trends in patient characteristics and risk factors

**Figure 3.**
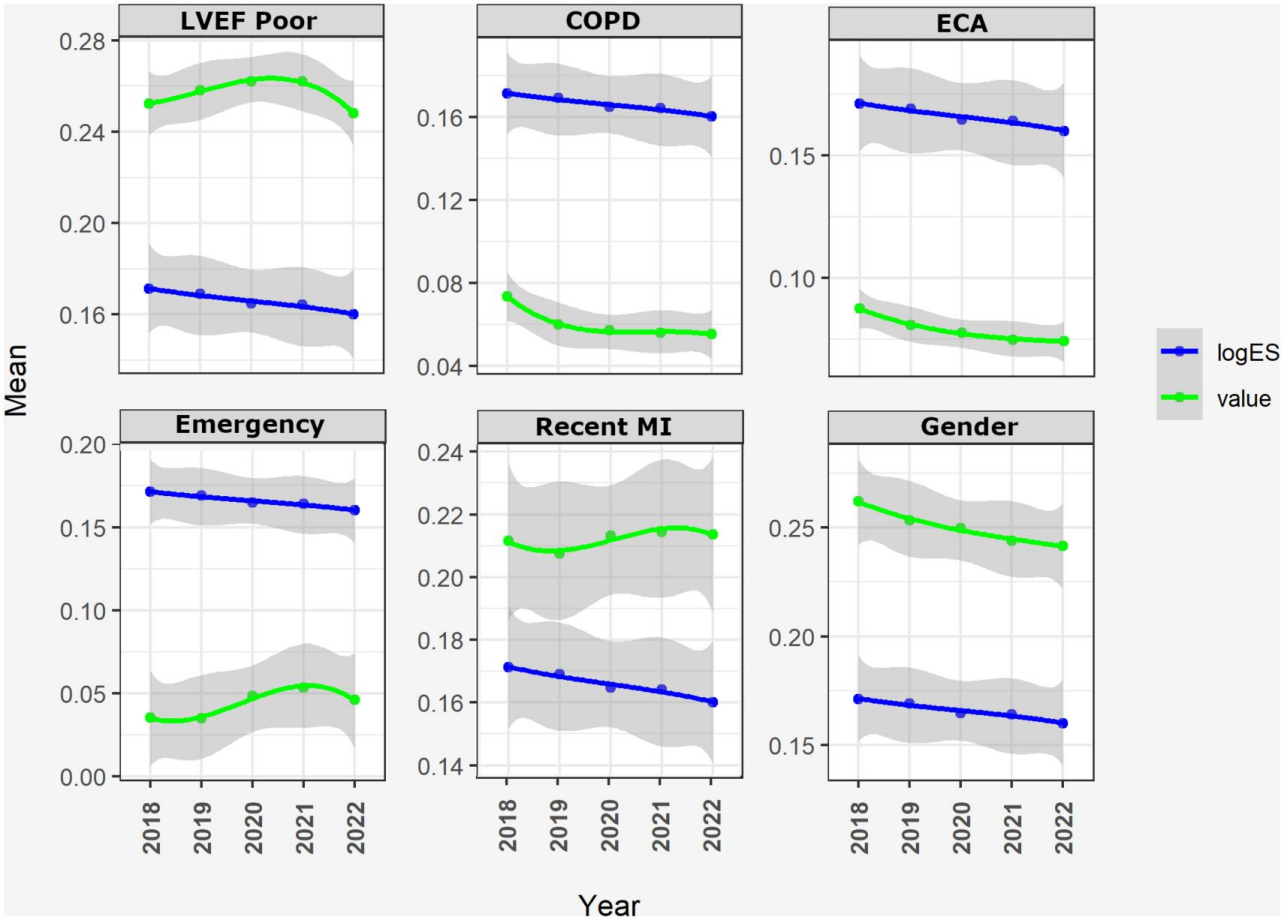
Change in Patients’ Logistic EuroSCORE Risk and Corresponding Normalized Risk Factors. 95% Confidence intervals are shown. ECA, extracardiac arteriopathy

The trends in patient characteristics across the 5 periods of the study are provided in **Table 1**. There was a rise in the proportion of emergency operations linked to pre-operative myocardial infarctions and left ventricular dysfunction during the pandemic period (**Figure 3**). The logistic EuroSCORE (logES) showed a slight decreasing trend in surgical mortality risk during the study period, which may be related to the decrease in the proportion of high-risk operations, female patients with chronic obstructive pulmonary disease (COPD) or extracardiac arteriopathy. There was little to no change in other LogES risk factors.

**Table 1.** Characteristics of Patients Undergoing Cardiac Surgery across the 5 Periods

	Overall	Pre-Lockdown	1st lockdown start to relaxation	2nd lockdown start to relaxation	3rd lockdown start to relaxation	Post lockdown	*P*-values
*N* ^a^	91 370	52 995	10 780	2975	8735	15 760	
Age (yr), median [IQR]	68.36 [59.81, 74.86]	68.88 [60.15, 75.23]	67.92 [59.52, 74.40]	67.35 [58.90, 74.04]	67.29 [59.08, 74.36]	67.63 [59.57, 74.48]	.006
Gender (Females) (%)	23 055 (25.3)	13 655 (25.8)	2755 (25.6)	678 (22.8)	2175 (24.9)	3790 (24.0)	<.001
IMD 2019 Quintiles (%)							.972
1	15 815 (17.4)	9174 (17.3)	1860 (17.3)	500 (16.8)	1545 (17.7)	2735 (17.4)	
2	17 613 (19.3)	10 163 (19.2)	2085 (19.4)	570 (19.2)	1720 (19.7)	3080 (19.5)	
3	19 169 (21.0)	11 156 (21.1)	2260 (21.0)	650 (22.0)	1810 (20.8)	3290 (20.9)	
4	19 489 (21.4)	11 328 (21.4)	2320 (21.6)	640 (21.5)	1810 (20.8)	3390 (21.5)	
5	19 041 (20.9)	11 091 (21.0)	2240 (20.8)	610 (20.5)	1840 (21.1)	3260 (20.7)	
Urgency (%)							<.001
Elective	51 273 (56.2)	32 385 (61.1)	5735 (53.2)	1530 (51.5)	3915 (44.8)	7705 (48.9)	
Urgent	36 161 (39.6)	18 745 (35.4)	4540 (42.1)	1270 (42.8)	4310 (49.4)	7295 (46.3)	
Emergency	3340 (3.7)	1631 (3.1)	440 (4.1)	145 (4.9)	440 (5.1)	680 (4.3)	
Salvage	450 (0.5)	225 (0.4)	60 (0.5)	25 (0.8)	60 (0.7)	80 (0.5)	
Creatinine at surgery (median [IQR])	84.00 [72.00, 99.00]	84.00 [73.00, 99.00]	83.00 [72.00, 98.00]	85.00 [73.00, 101.00]	83.00 [71.00, 98.00]	84.00 [72.00, 98.00]	.330
Hypertension (%)	62 647 (69.2)	36 223 (69.0)	7537 (70.3)	2023 (68.4)	6037 (69.5)	10 827 (69.1)	.296
Renal failure (dialysis dependent) (%)							.812
None	88 426 (98.4)	51 587 (98.4)	10 543 (98.6)	2885 (98.3)	8338 (98.3)	15 073 (98.4)	
Dialysis for acute renal failure	424 (0.5)	249 (0.5)	50 (0.5)	13 (0.4)	47 (0.6)	65 (0.4)	
Dialysis for chronic renal failure	626 (0.7)	369 (0.7)	63 (0.6)	25 (0.9)	58 (0.7)	111 (0.7)	
No dialysis but pre-operative acute renal failure (anuria or oliguria < 10 mL/hr)	349 (0.4)	204 (0.4)	33 (0.3)	11 (0.4)	36 (0.4)	65 (0.4)	
Neurological dysfunction (%)	2531 (2.8)	1590 (3.1)	265 (2.5)	85 (2.9)	221 (2.6)	370 (2.4)	<.001
Impaired mobility (%)	3978 (4.9)	2263 (5.1)	492 (4.8)	129 (4.6)	442 (5.2)	652 (4.2)	<.001
Interval surgery and last MI (%)							<.001
No previous MI	70 103 (77.8)	41 688 (79.5)	8362 (78.3)	2262 (76.9)	6286 (73.2)	11 505 (74.3)	
MI < 6 hours	262 (0.3)	153 (0.3)	33 (0.3)	10 (0.3)	28 (0.3)	38 (0.2)	
MI 6-24 hours	528 (0.6)	267 (0.5)	67 (0.6)	25 (0.9)	69 (0.8)	100 (0.6)	
MI 1-30 days	16 924 (18.8)	9075 (17.3)	2011 (18.8)	551 (18.7)	1922 (22.4)	3365 (21.7)	
MI 31-90 days	2296 (2.5)	1234 (2.4)	213 (2.0)	93 (3.2)	280 (3.3)	476 (3.1)	
Recent MI (%)	19 288 (21.1)	11 105 (21.0)	2277 (21.1)	657 (22.1)	1841 (21.1)	3408 (21.6)	0.295
NYHA (%)							<.001
I	16 670 (18.8)	9824 (19.0)	1982 (18.9)	577 (20.1)	1567 (18.5)	2720 (17.8)	
II	37 972 (42.8)	22 702 (44.0)	4225 (40.3)	1217 (42.3)	3475 (41.1)	6353 (41.7)	
III	28 264 (31.9)	16 125 (31.2)	3553 (33.9)	890 (30.9)	2712 (32.1)	4984 (32.7)	
IV	5822 (6.6)	3002 (5.8)	735 (7.0)	193 (6.7)	706 (8.3)	1186 (7.8)	
Angina status pre-surgery (%)	8797 (9.6)	5034 (9.5)	1038 (9.6)	295 (9.9)	868 (9.9)	1562 (9.9)	.451
Ejection fraction category (%)							<.001
Good	65 052 (72.5)	38 592 (73.5)	7669 (72.8)	2106 (72.0)	5967 (70.2)	10 718 (70.0)	
Poor	23 964 (26.7)	13 539 (25.8)	2780 (26.4)	786 (26.9)	2436 (28.7)	4423 (28.9)	
Very Poor	741 (0.8)	357 (0.7)	91 (0.9)	32 (1.1)	98 (1.2)	163 (1.1)	
Diabetes (%)							.021
Not diabetic	66 670 (73.8)	38 985 (74.2)	7859 (73.3)	2212 (74.8)	6267 (72.7)	11 347 (73.0)	
Diet	3615 (4.0)	2083 (4.0)	405 (3.8)	116 (3.9)	358 (4.2)	653 (4.2)	
Oral therapy	14 568 (16.1)	8346 (15.9)	1774 (16.6)	466 (15.8)	1435 (16.7)	2547 (16.4)	
Insulin	5518 (6.1)	3113 (5.9)	681 (6.4)	164 (5.5)	558 (6.5)	1002 (6.4)	
Pre-operative ventilation (%)	828 (0.9)	430 (0.8)	103 (1.0)	46 (1.5)	100 (1.1)	149 (0.9)	<.001
Pre-operative inotropic support (%)	1219 (1.3)	622 (1.2)	158 (1.5)	59 (2.0)	148 (1.7)	232 (1.5)	<.001
Active endocarditis (%)	2232 (2.4)	1216 (2.3)	273 (2.5)	72 (2.4)	247 (2.8)	424 (2.7)	.005

Continuous variables are in median and interquartile range; categorical variables are in frequency and percentage (%); Index of Multiple Deprivation (IMD); Myocardial infarction (MI); New York Heart Association (NYHA) score.

a *N* counts rounded to the nearest 5 according to NHS England’s output protocol.

### Early clinical outcomes

The trends in early post-operative outcomes across the five periods are provided in in **Table 2**. The in-hospital mortality, which was 2.32% pre-pandemic, was the highest during the first lockdown phase (2.88%) and continued to decrease during the second (2.7%) and third lockdown phases (2.61%). During the post-lockdown recovery phase, the mortality further reduced (2.52%) but was still higher than the pre-pandemic mortality. The number of patients requiring cardiac and respiratory support remained unchanged during the study periods. There was also no change in the rate of stroke, return to theatre for bleeding, deep wound infection, length of intensive therapy unit (ITU), or post-operative hospital stay (**Table 2**).

**Table 2.** Post-Operative Outcomes of Patients Undergoing Cardiac Surgery across the 5 Periods in the Study

	Overall	Pre-lockdown	1st lockdown start to relaxation	2nd lockdown start to relaxation	3rd lockdown start to relaxation	Post lockdown	*P*-value
N^a^	91 370	52 995	10 780	2975	8735	15 760	
Deep sternal wound infection (%)	514 (0.7)	314 (0.7)	52 (0.6)	14 (0.6)	52 (0.7)	82 (0.6)	.515
Post-op stroke (%)							.250
None	86 208 (97.8)	50 058 (97.8)	10 185 (97.8)	2823 (97.7)	8249 (97.6)	14 893 (98.1)	
Transient ischaemic attack	609 (0.7)	355 (0.7)	65 (0.6)	25 (0.9)	66 (0.8)	98 (0.6)	
Stroke	1288 (1.5)	753 (1.5)	169 (1.6)	41 (1.4)	133 (1.6)	192 (1.3)	
Post-op length of stay (days) (median [IQR])	7 [5, 10]	7 [5, 10]	7 [5, 10]	7 [5, 10]	7 [5, 10]	7 [5, 10]	.125
Return for bleeding (%)	2837 (3.10)	1667 (3.15)	344 (3.19)	103 (3.46)	265 (3.03)	458 (2.91)	.398
Cumulative bypass time (median [IQR])	97.00 [74.00, 129.00]	96.00 [73.00, 126.00]	98.00 [75.00, 130.00]	101.00 [77.00, 134.00]	101.00 [78.00, 134.00]	100.00 [77.00, 133.00]	.792
Length of intensive treatment unit stay (median [IQR])	4.00 [3.00, 6.00]	4.00 [3.00, 6.00]	4.00 [3.00, 6.00]	4.00 [3.00, 6.00]	4.00 [3.00, 6.00]	4.00 [3.00, 6.00]	.110
In-hospital mortality (%)	2249 (2.46)	1232 (2.32)	310 (2.88)	82 (2.76)	228 (2.61)	397 (2.52)	<.001
30-day mortality (%)	3082 (3.4)	434 (4.0)	1209 (7.7)	714 (1.3)	167 (5.6)	558 (6.4)	<.001
Advanced respiratory support (days) (median [IQR])	1 [1, 2]	1 [1, 2]	1 [1, 2]	1 [1, 2]	1 [1, 2]	1 [1, 2]	.042
Advanced cardiac support (days) (median [IQR])	2 [1, 4]	2 [1, 4]	2 [1, 4]	2 [1, 4]	2 [1, 4]	2 [1, 4]	.225
Total length of hospital stay (days) (median [IQR])	9 [7, 15]	9 [7, 15]	9 [7, 15]	10 [7, 15]	10 [7, 17]	10 [7, 17]	.006

Continuous variables are in median and interquartile range; categorical variables are in frequency and percentage (%).

a *N* counts rounded to the nearest 5 according to NHS England’s output protocol.

### Mid-term outcomes

**Figure 4.**
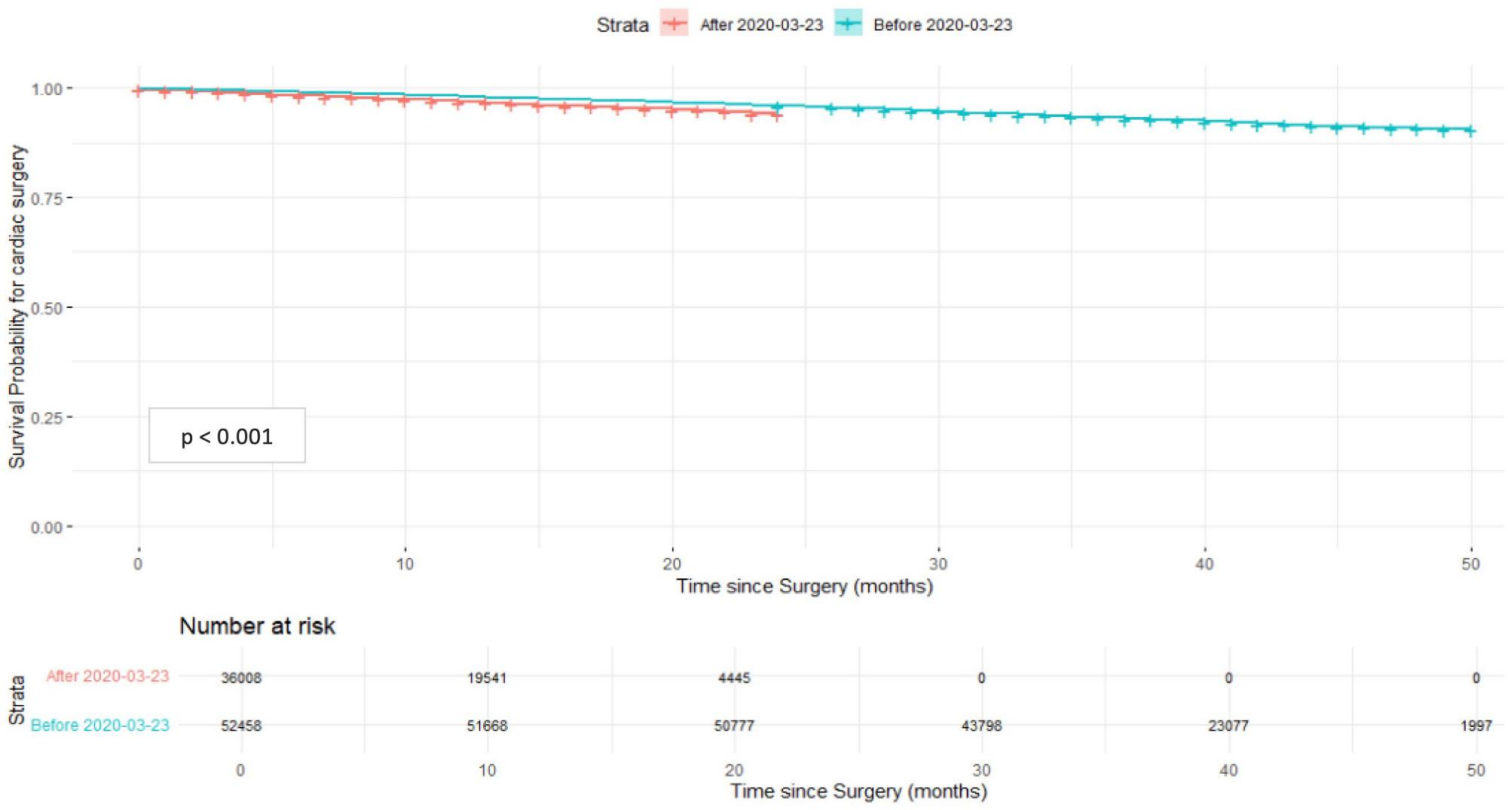
Changes in Mid-Term Outcomes in Terms of Survival Till End of Study (March 31, 2022). Number at risk are provided across strata and time periods. *P*-values are shown to 3 decimal places

Kaplan-Meier survival curves show a divergence occurring over time with those operated before the pandemic (March 2020) consistently maintaining a higher survival probability compared to those operated after (log-rank test *P*-value < .0001; **Figure 4**).

Adjusted Cox proportional hazard regression with confounders adjusted against the timing of operation also showed that patients who had surgery before March 23, 2020, had a significantly lower hazard of mortality (HR, 0.638; 95% CI, 0.5875-0.6921; **Table 3**). More specifically, the logistic regression with adjustment for logistic EuroSCORE in addition to age (quartiles), COVID diagnosis and hospital random effects revealed the significant increase in mortality risk to be attributed to the third lockdown period (OR, 1.09; 95% CI, 1.01-1.17; *P* < .05; **Figure S5**, bottom), though this significance is otherwise undetected without the adjustment for the logistic EuroSCORE. From the logistic regression comparing elective vs urgent, emergency and salvage groups, it can indeed be seen that there are significantly increased odds of urgent, emergency and salvage procedures compared to elective procedures in pandemic periods compared to the pre-lockdown period, with the third lockdown period having the highest relative difference in odds (OR, 2; 95% CI, 1.91-2.1, *P* < .05; **Figure S5**, top).

**Table 3.** Adjusted Cox Proportional Hazard Regression With and Without Confounders Adjustment for Patients Operated Before and After March 23, 2020

	HR (95% CI)	*P*-value
**Adjusted**		
Before March 23, 2020	0.638 (0.5882, 0.6930)	<.001
Age at operation	0.997 (0.9941, 0.9996)	.024
Female gender	0.928 (0.8649, 0.9965)	.040
Quintiles of IMD (Reference: Q1—most disadvantaged)		
Q2	1.004 (0.9111, 1.1060)	.938
Q3	1.012 (0.9194, 1.1137)	.809
Q4	1.003 (0.9111, 1.1050)	.945
Q5—least disadvantaged	1.040 (0.9441, 1.1459)	.427
Diabetes mellitus (Reference: None)		
On diet control	1.028 (0.8851, 1.1949)	.715
Oral medication	1.035 (0.9533, 1.1227)	.416
Insulin	0.933 (0.8189, 1.0622)	.293
Hypertension	0.990 (0.9467, 1.0342)	.640
Chronic obstructive pulmonary disease (COPD)	0.979 (0.9029, 1.0606)	.598
Extracardiac arteriopathy	1.084 (0.9767, 1.2035)	.129
Left ventricular ejection fraction (Reference: Good ≥ 50%)		
Poor 21%-30%	0.982 (0.9177, 1.0518)	.611
Very Poor < 21%	1.136 (0.8233, 1.5667)	.438
Surgery type (Reference: CABG only)		
Aortic valve procedure only	0.958 (0.8823, 1.0406)	.310
Mitral valve procedure only	0.938 (0.8315, 1.0574)	.294
Other procedure only	0.820 (0.6615, 1.0159)	.069
CABG and aortic value procedure	0.887 (0.7922, 0.9926)	.037
CABG and mitral valve procedure	0.930 (0.7498, 1.1541)	.511
CABG and other procedure	1.179 (0.9410, 1.4770)	.152
Other combination	0.992 (0.8846, 1.1133)	.896
Operative urgency (Reference: elective)		
Urgent	1.051 (0.9854, 1.1200)	.131
Emergency	1.022 (0.8651, 1.2085)	.794
Salvage	1.052 (0.6665, 1.6592)	.829
Cardiopulmonary bypass time	1.001 (1.0001, 1.0010)	.021
**Unadjusted**		
Before March 23, 2020	0.641 (0.5929, 0.6922)	<.001

Abbreviation: CABG, coronary artery bypass grafting.

The variations in reasons for re-admission show that although re-admission due to hypertension, and cerebrovascular diseases in the recovery phase had decreased, there was an increase in readmissions due to valve-related issues.

## DISCUSSION

The findings of this study clearly demonstrate the profound impact of the COVID-19 pandemic on adult cardiac surgical practice in the United Kingdom. Importantly, the mortality risk was lower before the pandemic (March 23, 2020) and was higher after this point, and this can be specifically attributed to an increase in mortality risk in the third lockdown period. This to a large extent reflects the significantly increased likelihood of urgent, emergency and salvage procedures being performed compared to elective procedures in pandemic periods compared to the pre-pandemic period. The stark decline in surgical volume during lockdown periods, coupled with the incomplete recovery observed even after the easing of restrictions, paints a picture of a system strained to its limits during COVID and in common with other parts of the health service, one that has not fully recovered. Despite the drop in numbers in all categories, there was a surge in the volume of cases done urgently, driven by stoppages and substantial delays in elective care.

Waiting times for elective surgery in the UK NHS were already rising prior to the pandemic.^14^ However, the significant increase in waiting times in the pandemic periods compared to that of the pre-pandemic period for elective and urgent procedures raises additional concerns for patient health care and in terms of the potential for disease progression. The drop observed during the pandemic in operative risk as measured by the lower EuroSCOREs suggests some degree of case selection, possibly driven by the need to avoid cases that may have required prolonged ITU stays. Despite this drop, the persistently higher early and more worryingly late mortality, even after adjusting for covariates in the post-pandemic period, raises concerns about operative risks that are not picked up by standard risk scoring systems. These may be related to undiagnosed incident illness or late effects of infection with the coronavirus. This increase in mid-term mortality is also likely to be a long-term consequence of delayed surgery and disrupted follow-up care. Higher post-pandemic national mortality rates have been previously noted.^15^ The effects of lockdown on the provision of healthcare and the continued difficulty of accessing primary care has had dramatic negative effects on the cardiovascular health of the nation as well as the health of the NHS. Surprisingly, we did not find any other difference in the rate of stroke, return to theatre for bleeding, cardiovascular and respiratory support or ITU, and length of hospital stay. Furthermore, we did not find any clinically meaningful difference in the rate of cardiovascular-related readmissions.

Similar to other countries, such as Canada, the study here shows that the UK's cardiac surgery numbers decreased during the COVID-19 pandemic and have not fully returned to pre-pandemic levels.^16^ In addition, there is also agreement in the data that mortality rates have increased during the pandemic period.^16^ An important research question that this project has not answered is whether the findings of this study reflect outcomes from all cardiothoracic centres across the United Kingdom. While this appears likely to be the case, it is possible that individual centres may have devised ways of working to mitigate the impact of the pandemic.

There is evidence that the incomplete recovery of cardiac surgical activity, as revealed in the national picture, is not reflected across the nation.^17^ There is a great degree of heterogeneity with larger centres being able to recover to and exceed their pre-pandemic levels of activity much earlier than smaller centres many of which still languish behind,^17^ seemingly lacking the capacity to recover completely.

The COVID-19 pandemic has left a lasting imprint on cardiac surgery in the United Kingdom, with reduced capacity, increased urgency, and prolonged waiting times. Moving forward, proactive strategies are needed to address the surgical backlog and mitigate the long-term consequences of delayed care. This may include expanded, possibly centralized capacity, innovative models of service delivery, and personalized interventions to support high-risk patients.

In conclusion, as we slowly emerge from this turbulent period in history, recovery of elective activity, ageing of the population, and increasing diagnostic ability should mean that the need for surgical treatment of structural cardiac disease, with its durable effect on prognosis and limiting rehospitalization, should be a priority. Continued surveillance and monitoring of activity and outcomes will be vital to ensure that lessons learned during the pandemic are translated into a more resilient and responsive cardiac surgical service for the future.

### Limitations and future work

One limitation of this work is that there was no prespecified plan to adjust for multiple comparisons. Future work should aim to provide more stringent control for false-positive discovery rates. Non-ST-elevation myocardial infarction (NSTEMI) and ST-elevation myocardial infarction (STEMI) information were not available in the dataset.

## Supplementary Material

ezaf246_Supplementary_Data

## Data Availability

The data availability statements are provided in the Supplementary Materials, Data Availability section.
