# Supplementary material for: Cardiac Surgery During and After the Pandemic: A Retrospective Analysis of UK Trends and Outcomes
Source: Eur J Cardiothorac Surg. 2025 Jul 28;67(8):ezaf246. doi: 10.1093/ejcts/ezaf246 (PMC12371327; doi:10.1093/ejcts/ezaf246)
Supplement: ezaf246_Supplementary_Data [file ezaf246_supplementary_data.zip › Supplementary Materials_v1_5.docx]

**Supplementary Materials**

**Covariates**

Covariates were defined as follows: primary diagnosis was determined by linking NACSA records with HES APC records that had admission dates within a 7-day window of the surgery, monthly cardiac admissions were calculated for the six months preceding the operation date, using 30-day periods for each month, angiogram and cardiac catheterization dates were supplemented using HES APC and HES OP records within 18 months prior to the operation date, HES Critical Care records provided data on the number of critical care unit admissions and length of stay during the index admission, as well as details on the number of days for which patients required various types of respiratory and cardiac support; all other covariates were defined in the NACSA dataset. Non-elective was defined separately from elective as urgent, emergency and salvage. Non-CABG procedure was defined as any procedure that was not isolated CABG.

**Dealing with missingness**

Data processing of data variables were conducted as per NICOR NACSA audit data cleaning protocol using R v4.0.2. Missing data for baseline information was very low (<1%). However, since no prediction modelling is considered here, missing values were not imputed, such that only complete cases of data were used in model analysis step.

| **ICD-10** | **Disease** | **Type** | **Sub-classification** |
| --- | --- | --- | --- |
| Total number readmitted |  |  |  |
| I10-I15 Hypertensive disease | | | |
| I10 | Essential (primary) hypertension | Cardiovascular | Hypertensive disease |
| I11 | Hypertensive heart disease | Cardiovascular | Hypertensive disease |
| I12 | Hypertensive renal disease | Cardiovascular | Hypertensive disease |
| I13 | Hypertensive heart and renal disease | Cardiovascular | Hypertensive disease |
| I15 | Secondary hypertension | Cardiovascular | Hypertensive disease |
| I20-I25 Ischaemic Heart Disease | | | |
| I20 | Angina pectoris | Cardiovascular | Ischaemic heart disease |
| I21 | Acute myocardial infarction | Cardiovascular | Ischaemic heart disease |
| I22 | Subsequent myocardial infarction | Cardiovascular | Ischaemic heart disease |
| I23 | Certain current complications following acute myocardial infarction | Cardiovascular | Ischaemic heart disease |
| I24 | Other acute ischaemic heart diseases | Cardiovascular | Ischaemic heart disease |
| I25 | Chronic ischaemic heart disease | Cardiovascular | Others |
| I26-I28 Pulmonary heart disease and diseases of pulmonary circulation | | | |
| I26 | Pulmonary embolism | Cardiovascular | Pulmonary embolism |
| I27 | Other pulmonary heart diseases | Cardiovascular | Others |
| I28 | Other diseases of pulmonary vessels | Cardiovascular | Others |
| I30-I52 Other forms of heart disease | | | |
| I30 | Acute pericarditis | Cardiovascular | Valve related disorders |
| I31 | Other diseases of pericardium | Cardiovascular | Valve related disorders |
| I32 | Pericarditis in diseases classified elsewhere | Cardiovascular | Valve related disorders |
| I33 | Acute and subacute endocarditis | Cardiovascular | Valve related disorders |
| I34 | Nonrheumatic mitral valve disorders | Cardiovascular | Valve related disorders |
| I35 | Nonrheumatic aortic valve disorders | Cardiovascular | Valve related disorders |
| I36 | Nonrheumatic tricuspid valve disorders | Cardiovascular | Valve related disorders |
| I37 | Pulmonary valve disorders | Cardiovascular | Valve related disorders |
| I38 | Endocarditis, valve unspecified | Cardiovascular | Valve related disorders |
| I39 | Endocarditis and heart valve disorders in diseases classified elsewhere | Cardiovascular | Valve related disorders |
| I40 | Acute myocarditis | Cardiovascular | Others |
| I41 | Myocarditis in diseases classified elsewhere | Cardiovascular | Others |
| I42 | Cardiomyopathy | Cardiovascular | Others |
| I43 | Cardiomyopathy in diseases classified elsewhere | Cardiovascular | Others |
| I44 | Atrioventricular and left bundle-branch block | Cardiovascular | Arrhythmia |
| I45 | Other conduction disorders | Cardiovascular | Arrhythmia |
| I46 | Cardiac arrest | Cardiovascular | Others |
| I47 | Paroxysmal tachycardia | Cardiovascular | Arrhythmia |
| I48 | Atrial fibrillation and flutter | Cardiovascular | Arrhythmia |
| I49 | Other cardiac arrhythmias | Cardiovascular | Arrhythmia |
| I50 | Heart failure | Cardiovascular | Heart failure |
| I51 | Complications and ill-defined descriptions of heart disease | Cardiovascular | Others |
| I52 | Other heart disorders in diseases classified elsewhere | Cardiovascular | Others |
| I60-I69 Cerebrovascular diseases | | | |
| I60 | Subarachnoid haemorrhage | Cardiovascular | Cerebrovascular diseases |
| I61 | Intracerebral haemorrhage | Cardiovascular | Cerebrovascular diseases |
| I62 | Other nontraumatic intracranial haemorrhage | Cardiovascular | Cerebrovascular diseases |
| I63 | Cerebral infarction | Cardiovascular | Cerebrovascular diseases |
| I64 | Stroke, not specified as haemorrhage or infarction | Cardiovascular | Cerebrovascular diseases |
| I65 | Occlusion and stenosis of precerebral arteries, not resulting in cerebral infarction | Cardiovascular | Cerebrovascular diseases |
| I66 | Occlusion and stenosis of cerebral arteries, not resulting in cerebral infarction | Cardiovascular | Cerebrovascular diseases |
| I67 | Other cerebrovascular diseases | Cardiovascular | Cerebrovascular diseases |
| I68 | Cerebrovascular disorders in diseases classified elsewhere | Cardiovascular | Cerebrovascular diseases |
| I69 | Sequelae of cerebrovascular disease | Cardiovascular | Cerebrovascular diseases |
|  | | | |

Supplementary Table 1 ICD-10 diagnostic codes of the main cardiovascular reasons for readmission.


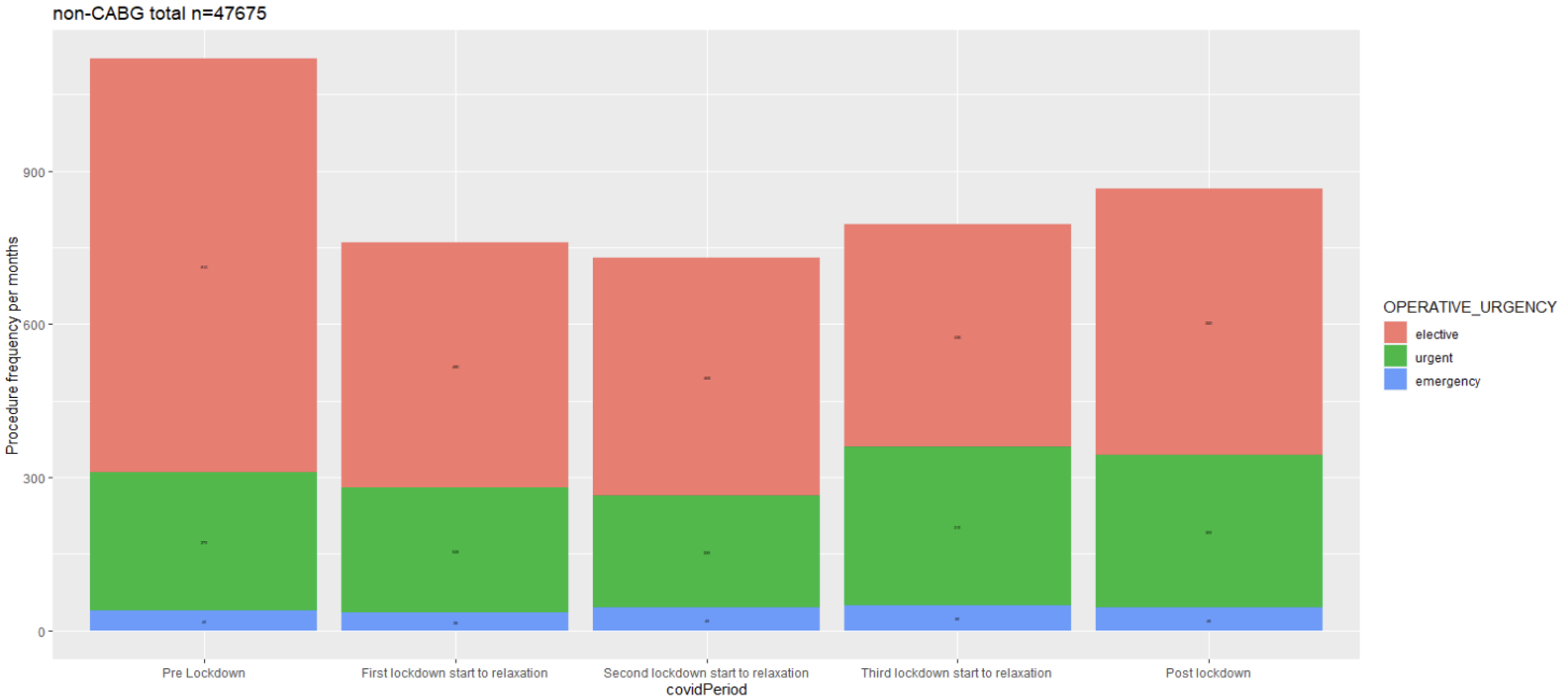


Supplementary figure 1*.* non-CABG procedures subdivided by: elective, urgent, emergency categories; y-axis represents the average number of procedures per months.


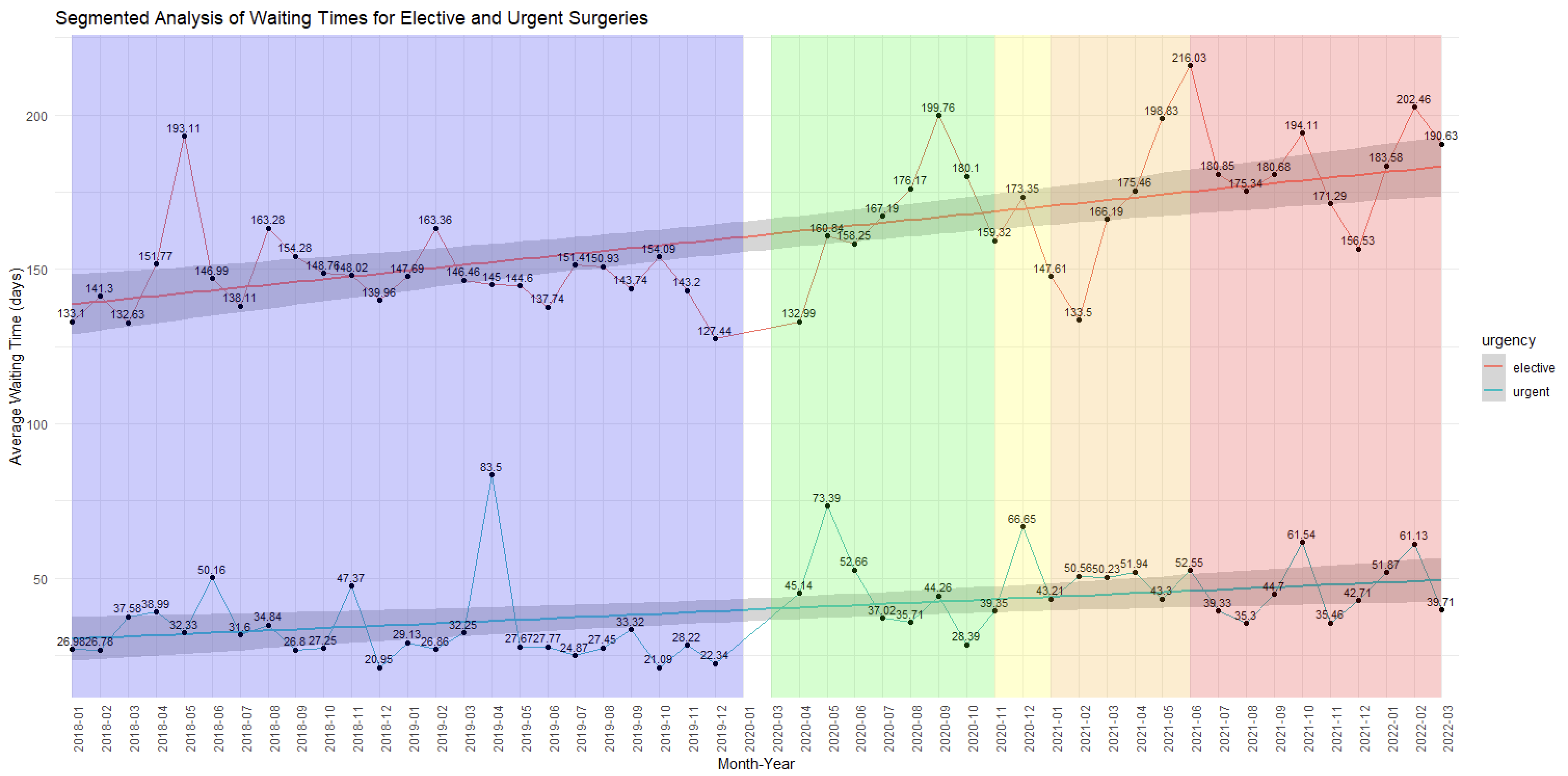


Supplementary figure 2 change in waiting time for both elective and urgent cardiac surgery - measured by time between last cardiac catheterisation and date of surgery; y-axis shows the average waiting time in days for each month.


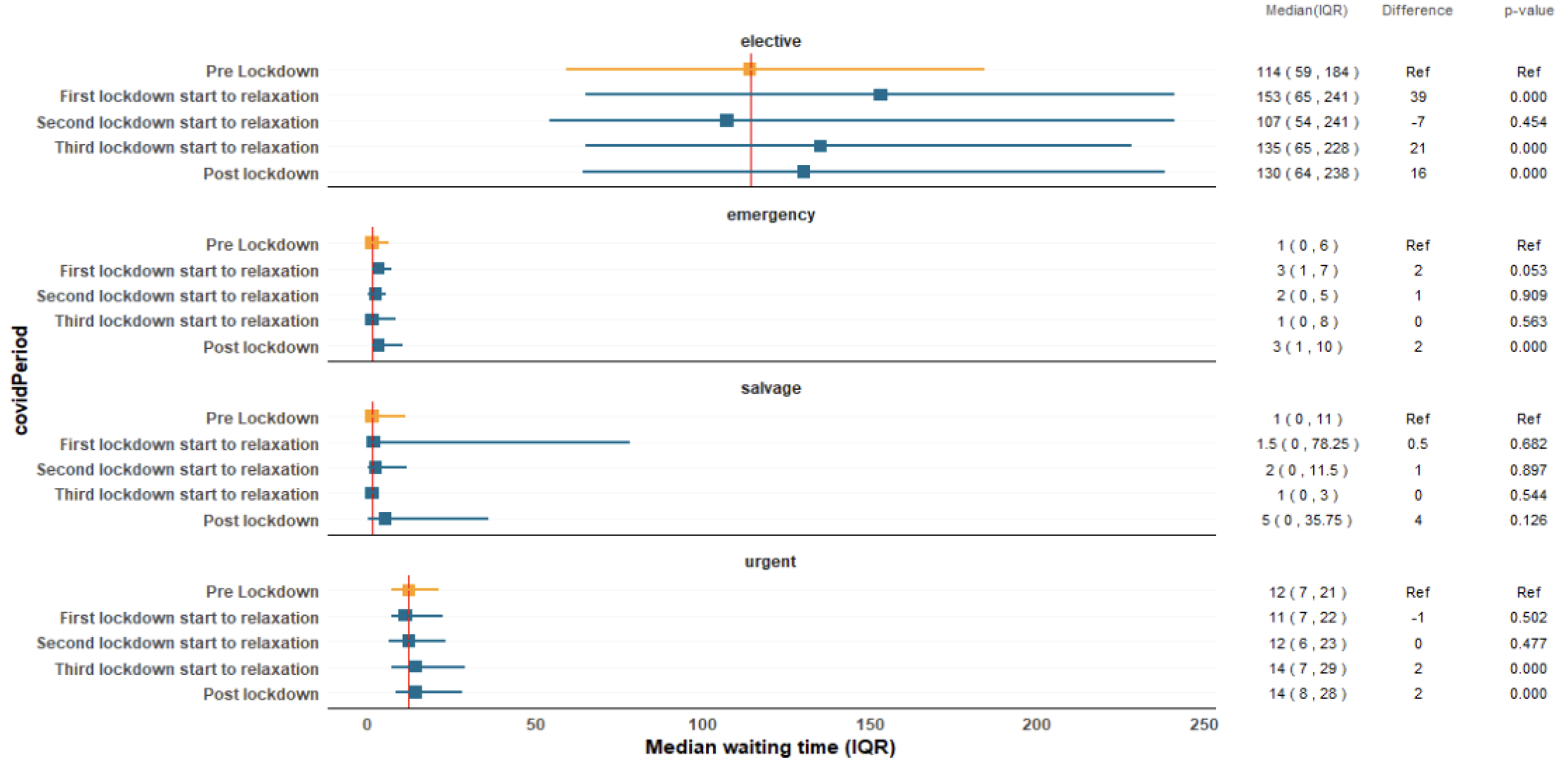


Supplementary figure 3 The difference in the waiting times of elective, urgent, emergency and salvage cardiac procedures comparing pre-lockdown period to other pandemic periods. Median (IQR) and difference values in median days per month are shown. P values (3 decimal places) for the difference between each pandemic period and the pre-lockdown period were calculated using the Wilcoxon rank sum test.


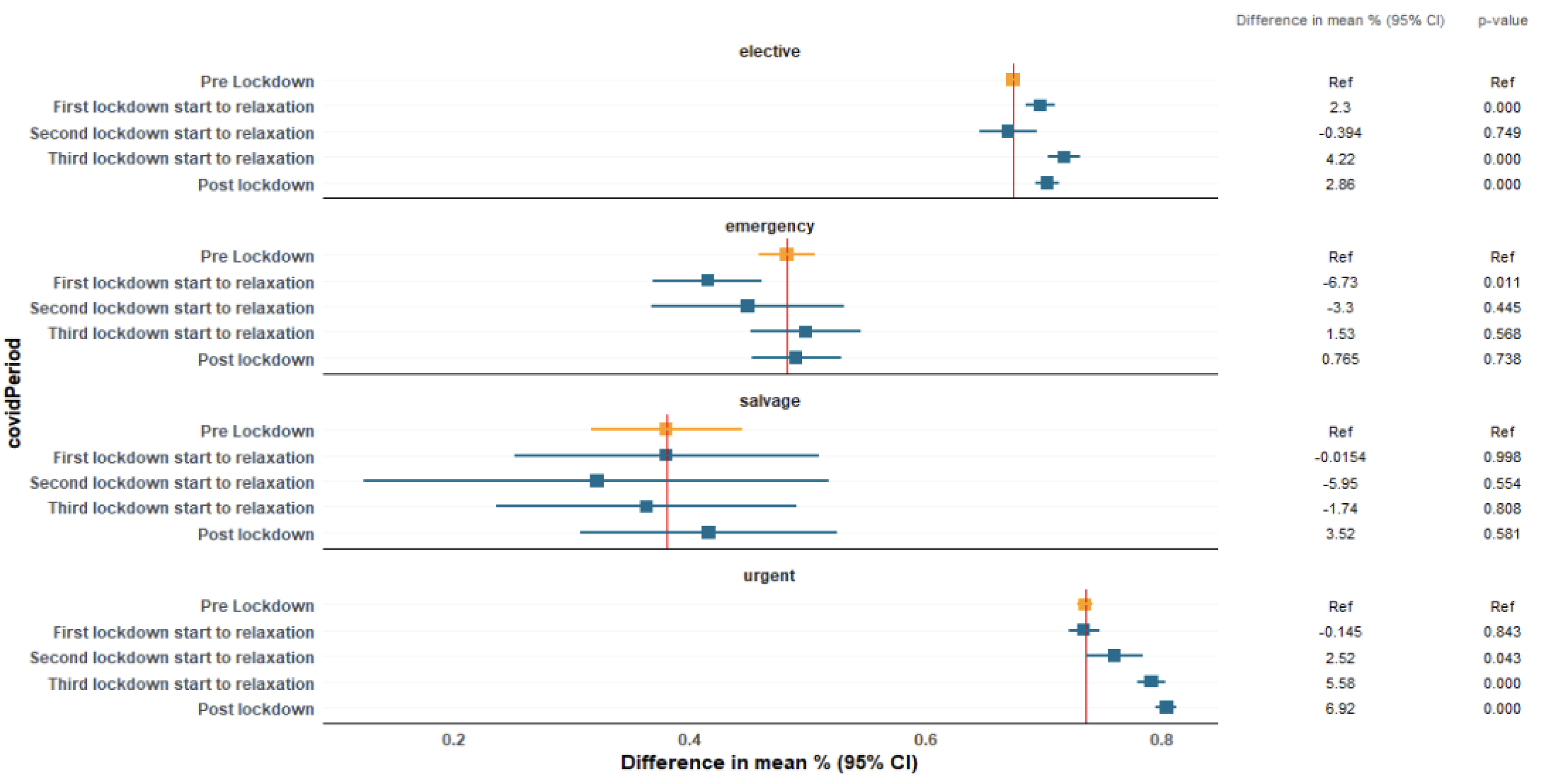


Supplementary figure 4 Difference in the mean percentage of each urgency group during pre-lockdown period compared to the other pandemic periods. Difference in mean percentage (95% CI) estimated in comparison to the pre-lockdown period, with p-values (3 decimal places) shown.


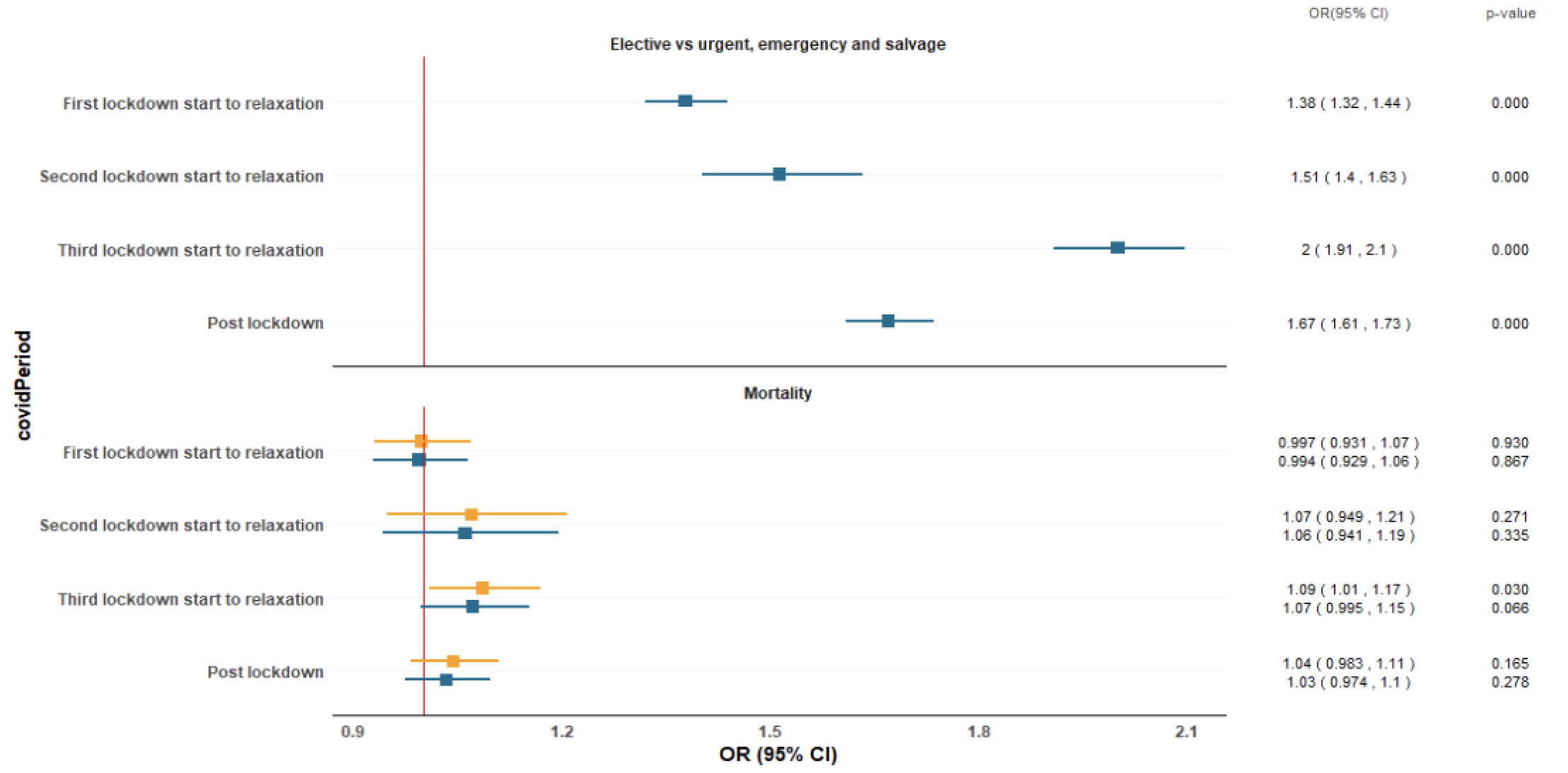


Supplementary figure 5 ORs of top: elective vs urgent, emergency and salvage groups and bottom: postprocedure mortality any time within specified pandemic periods (yes vs no). OR (95% CI) is estimated comparing pre-lockdown period to other pandemic periods. Results blue line: show the age (quartiles) and covid diagnosis adjusted mixed effects logistic regression ORs and orange line shows the ORs with additional adjustment for case mix using logistic EuroSCORE risk factors; p-values are shown to 3 decimal places.

**Ethical approval and information governance**

The North East – Newcastle and North Tyneside 2 research ethics committee provided ethical approval for the CVD-COVID-UK/COVID-IMPACT research programme (REC No 20/NE/0161) to access, within secure trusted research environments, unconsented, whole-population, de-identified data from electronic health records collected as part of patients’ routine healthcare.

**Funding**

The British Heart Foundation Data Science Centre (grant No SP/19/3/34678, awarded to Health Data Research (HDR) UK) funded co-development (with NHS England) of the Secure Data Environment service for England, provision of linked datasets, data access, user software licences, computational usage, and data management and wrangling support, with additional contributions from the HDR UK Data and Connectivity component of the UK Government Chief Scientific Adviser’s National Core Studies programme to coordinate national COVID-19 priority research. Consortium partner organisations funded the time of contributing data analysts, biostatisticians, epidemiologists, and clinicians.

**Data availability**

The data used in this study are available in NHS England’s Secure Data Environment (SDE) service for England, but as restrictions apply they are not publicly available (<https://digital.nhs.uk/services/secure-data-environment-service>). The CVD-COVID-UK/COVID-IMPACT programme, led by the BHF Data Science Centre (<https://bhfdatasciencecentre.org/>), received approval to access data in NHS England’s SDE service for England from the Independent Group Advising on the Release of Data (IGARD) (<https://digital.nhs.uk/about-nhs-digital/corporate-information-and-documents/independent-group-advising-on-the-release-of-data>) via an application made in the Data Access Request Service (DARS) Online system (DARS-NIC-381078-Y9C5K). The CVD-COVID-UK/COVID-IMPACT Approvals & Oversight Board (<https://bhfdatasciencecentre.org/areas/cvd-covid-uk-covid-impact/>) subsequently approved this project to access the data within NHS England’s SDE service for England. The de-identified data used in this study were made available to accredited researchers only. Those wishing to gain access to the data should contact [bhfdsc@hdruk.ac.uk](mailto:bhfdsc@hdruk.ac.uk) in the first instance.
